# Management of Complex Crown Fractures: A Case Series

**DOI:** 10.7759/cureus.37907

**Published:** 2023-04-20

**Authors:** Deepika Lakshmaiah, Visshnuvardhini SR, Sangita Ilango, Nikesh Sakthi, Sreelakshmi PS

**Affiliations:** 1 Conservative Dentistry and Endodontics, Chettinad Dental College and Research Institute, Chennai, IND

**Keywords:** maxillary anterior teeth, trauma, reattachment, crown fracture, complicated fracture

## Abstract

Maxillary teeth are most vulnerable to fracture due to trauma. An effective treatment plan for an anterior teeth fracture not only improves function and appearance but also benefits the patient psychologically. The reattachment of the fragmented tooth is one of the best treatment methods for such condition. It is considered as a better treatment option because it is uncomplicated, aesthetic, and preserves the dental structure. To achieve a positive prognosis, patient cooperation and awareness about the treatment are essential. This article includes three case reports that illustrate the management of complex maxillary anterior teeth fractures wherein the reattachment of the fractured tooth segments was undertaken.

## Introduction

Crown fractures of the anterior teeth are the most frequently noted type of dental trauma. The maxillary incisors are more vulnerable to crown fractures due to trauma owing to their location in the arch, whereas the mandibular central incisors are less commonly affected [1,2]. Crown fractures can be categorized as complicated and uncomplicated depending upon the involvement of the pulp [1]. Crown fractures of permanent incisors account for about 18%-22% of dental hard tissue injuries among which 28%-44% are uncomplicated and 11%-15% are complicated fractures [3]. As a result, there are limitations in function, aesthetics, and phonetics.

A fractured anterior tooth should be managed immediately since delayed treatment might result in periapical and pulpal pathologies. It can also induce a psychological pressure on the patient as a result of poor aesthetics [1]. During treatment planning, a number of factors must be taken into account, specially the patient's age, and the extent and position of the fracture line. Moreover, the biological width, alveolar bone fractures if any and damage to adjacent structures are also to be considered. The presence or absence of a fractured fragment, occlusion, aesthetics and financial considerations should also be taken into account to formulate a proper treatment plan [4].

Uncomplicated dental injuries are those that involve only enamel and dentin without the exposure of pulp [5]. The most commonly preferred course of treatment for such cases is to use aesthetic composite restoration to restore the lost tooth structure. Complicated crown fractures usually require root canal treatment combined with direct and indirect restorative techniques like composites, ceramic restorations and intra-radicular posts with crown placement [6].

The procedure of fragment reattachment was initially reported by Chosack and Eidelman in 1964 [7]. Later on, Tennery in 1978 reported the use of an acid-etching procedure to reattach the fragmented tooth segments [8]. If the natural tooth fragment is retained after fracture, then it can be reattached through adhesive procedures. This provides a favorable psychological reaction and acts as a biologic and aesthetic restoration. This case series describes three different modalities for management of complex crown fractures of maxillary anterior teeth.

## Case presentation

Case 1

**Figure 1:**
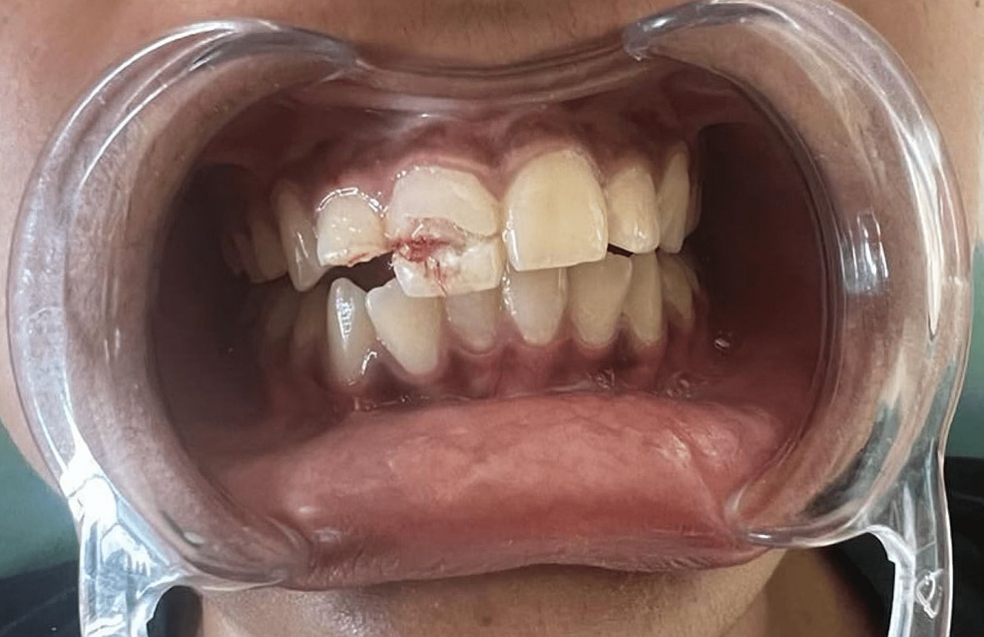
Pre-operative clinical photograph showing the enamel and dentin fracture with an evident pulpal exposure in 11 and 12

A 19-year-old female reported with a chief complaint of moderate pain and broken upper front teeth after 15 hours of impact due to fall from her vehicle. The clinical examination revealed a complex fracture in 11 (Ellis class 3) wherein the fracture was seen in the middle third extending mesio-distally (Figure 1). The fragment was not completely dislodged but had an evident enamel and dentin structure loss on the buccal aspect of 11. There was an Ellis and Davey class 3 fracture in 12 with no concomitant soft tissue damage. A normal probing depth was observed on examination. It was diagnosed as a complicated crown fracture in relation to the maxillary right central and lateral incisor. Her medical history was irrelevant. The patient was explained about the treatment options and informed consent was obtained.

**Figure 2:**
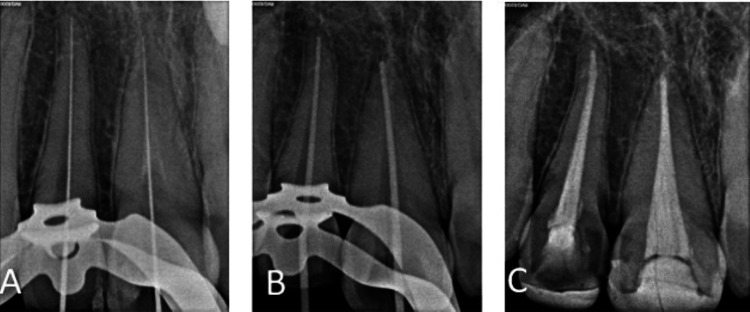
Intraoral periapical radiographs of 11, 12: (A) working length determination; (B) master cone placement; (C) post-operative radiograph after obturation and fragment stabilization

Local anesthesia (2% lidocaine containing adrenaline of 1:80,000) was given and rubber dam isolation was done. Shade matching was done prior to treatment initiation. The fractured segment was stabilized using a flowable composite (Tetric N-Ceram) both on the palatal and proximal surfaces. An access opening was initiated buccally and pulpal tissue was extirpated. Working length determination was done using an electronic apex locator (Root ZX Mini; J Morita Corp., Osaka) and was radiographically confirmed. Cleaning and shaping was done using K-files up to an apical size of 50. During preparation, irrigation was done with 5.25% sodium hypochlorite and 17% EDTA as a final irrigant. The root canal was dried using paper points. Finally, cold lateral compaction was done using gutta percha and sealer (zinc oxide eugenol sealer) (Figure 2).

**Figure 3:**
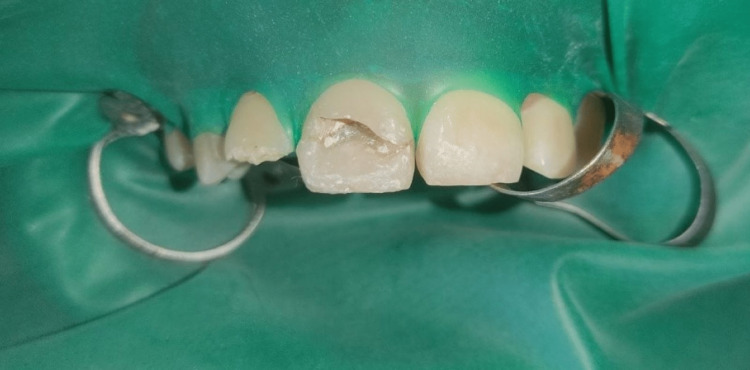
Fractured fragment stabilized in 11 using adhesive protocols

**Figure 4:**
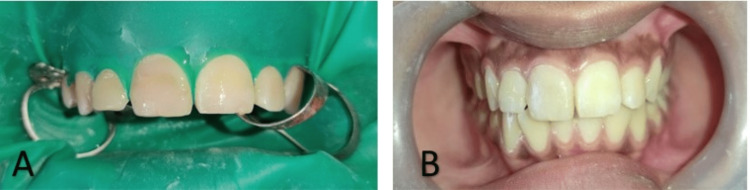
Post-operative clinical photographs of 11 and 12 taken (A) immediately and (B) at six months of follow-up

Glass ionomer cement (GIC) was applied as base. Tooth fragment and GIC were etched using 37% phosphoric acid (prime dental etchant) for 15 seconds followed by water rinsing for 10 seconds. Excess water was removed on gentle air drying. Bonding agent (3M ESPE single bond; St. Paul, MN) was applied to the etched surfaces using applicator tips in gentle brushing strokes. The excess bonding agent was removed and light cured for 20 seconds. A thin layer of the flowable composite was placed and light cured for 40 seconds (Figure 3). The incremental composite buildup was done to mimic the natural tooth structure. Then, final finishing and polishing of the teeth was done (Figure 4). Occlusion was examined, and the patient was given post-operative instructions. A post-operative radiograph was taken immediately. Follow-up was done for one, three and six months, respectively.

Case 2

**Figure 5:**
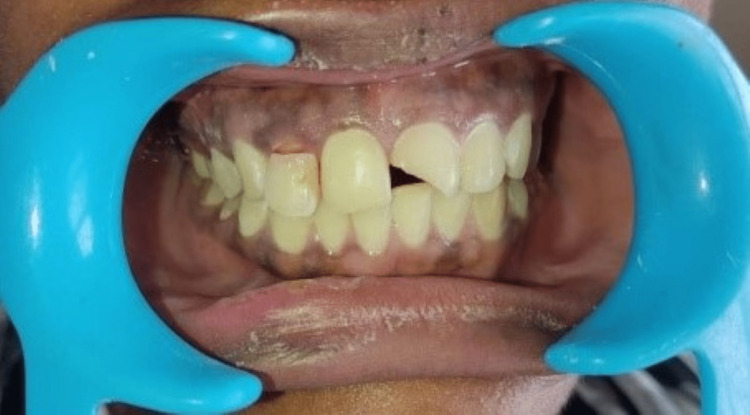
Pre-operative clinical photograph showing fractured 12 and 21

A 25-year-old female reported with broken upper front teeth after 18 hours of impact from a fall down the stairs. On clinical examination, an Ellis and Davey class 2 and 3 fracture was observed in 21 and 12, respectively (Figure 5). The fracture line was supra-gingival extending mesio-distally in 12. In relation to 21, there was an oblique fracture line in the mesio-distal aspect. It was planned to bond the fractured segment following an endodontic treatment in 12 after which a direct composite was proposed for 21. Informed consent was obtained from the patient prior to the procedure.

**Figure 6:**
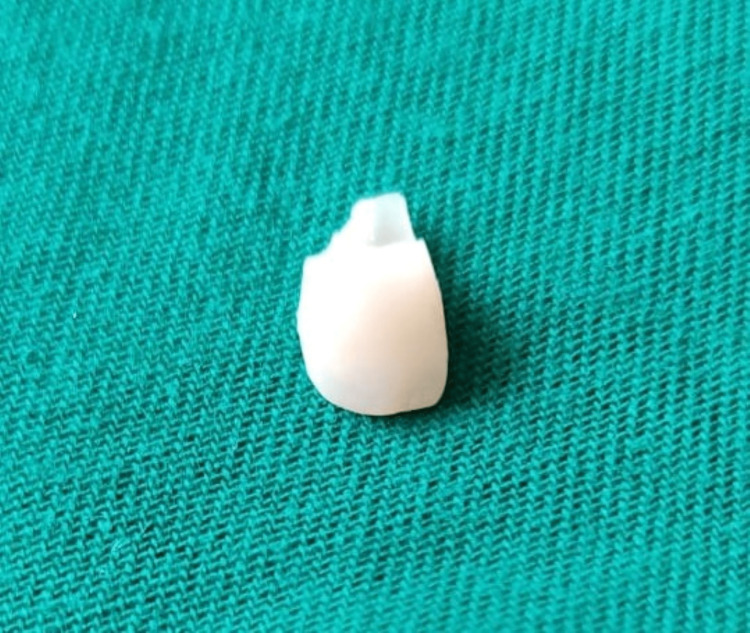
Removed tooth fragment of 12

**Figure 7:**
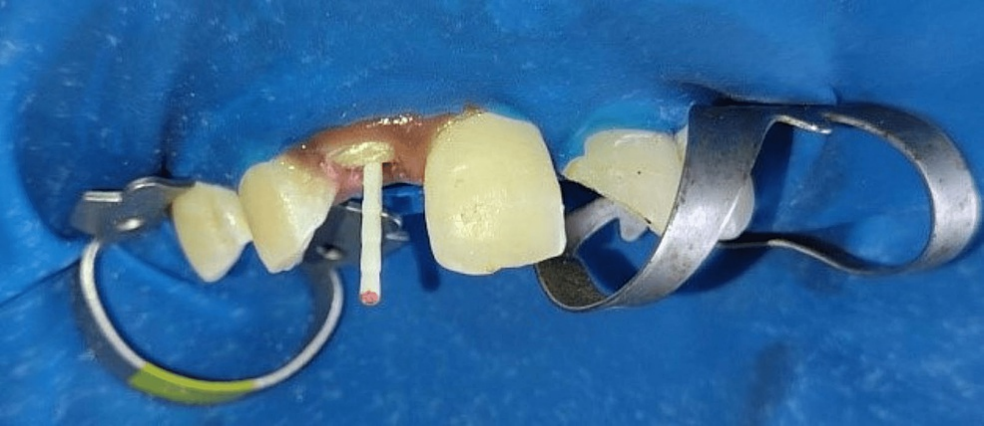
Placement of the fiber post after sectional obturation in 12

**Figure 8:**
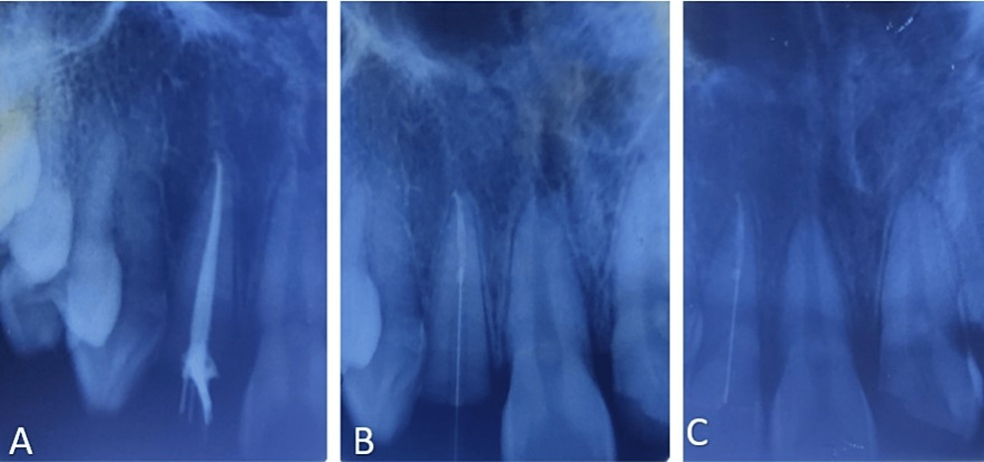
Intra-oral periapical radiographs depicting post placement and fragment reattachment in 12: (A) post-obturation radiograph, (B) placement of the fiber post, (C) after post placement and fragment reattachment in 12

**Figure 9:**
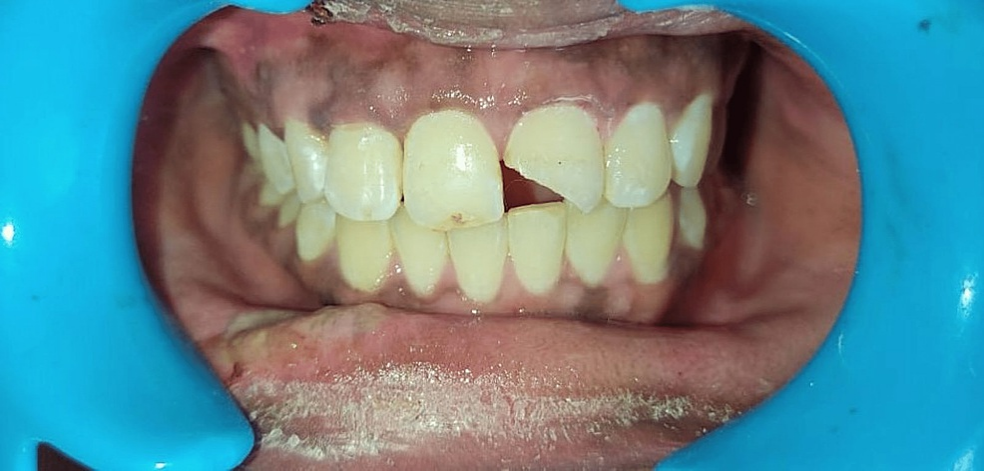
Post-operative clinical photograph after fragment reattachment in 12

**Figure 10:**
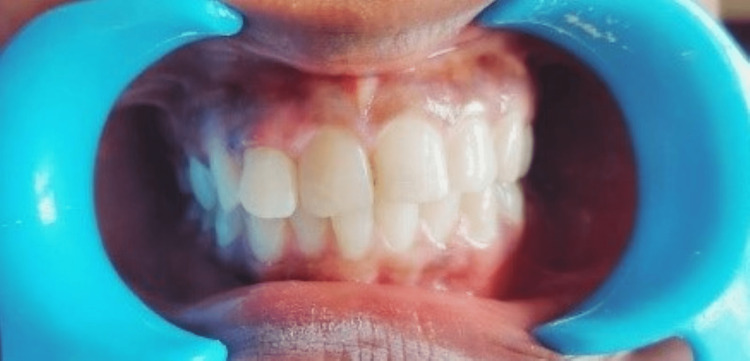
Clinical picture after a follow-up period of six months

Infiltration administration of the local anesthetic was done and rubber dam isolation was completed. The tooth fragment was removed gently and stored in saline (Figure 6). The root canal procedure was completed in a single visit. Obturation was done with gutta percha (using an AH plus sealer) following which post space preparation was done till Peeso reamer size 3. Etching of the fiber post (Reforpost; Angelus, Londrina, Brazil), root canal space and fractured segment was done with universal etchant (3M Scotchbond) for 15 seconds. Later, rinsing, and gentle air drying was done. Adhesive application (3M ESPE single bond) was done on both fiber post and the tooth (Figure 7). Dual cure resin (Prevest Fusion Ultra D/C; Prevest DenPro Limited, Jammu, India) was used for luting the post and the fractured segment was approximated with the flowable composite. A diamond finishing bur was used to remove the excess composite after curing. Direct composite restoration was done in the left central incisor. Thereafter, final finishing and polishing was done (Figures 8, 9). The patient was reviewed for one, three and six months (Figure 10).

Case 3

**Figure 11:**
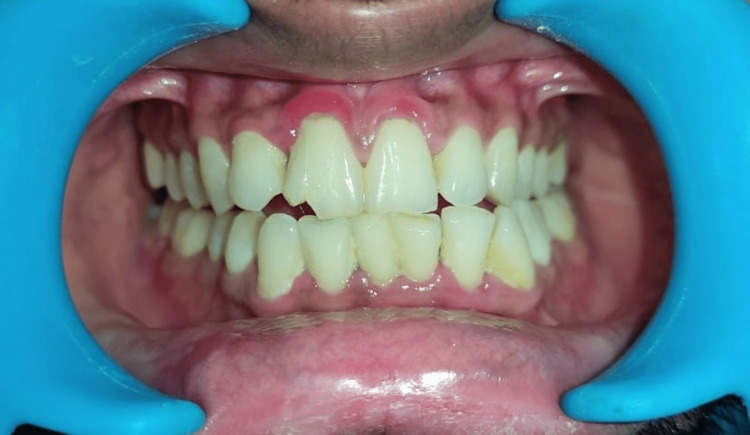
Pre-operative clinical photograph showing fractured 11

**Figure 12:**
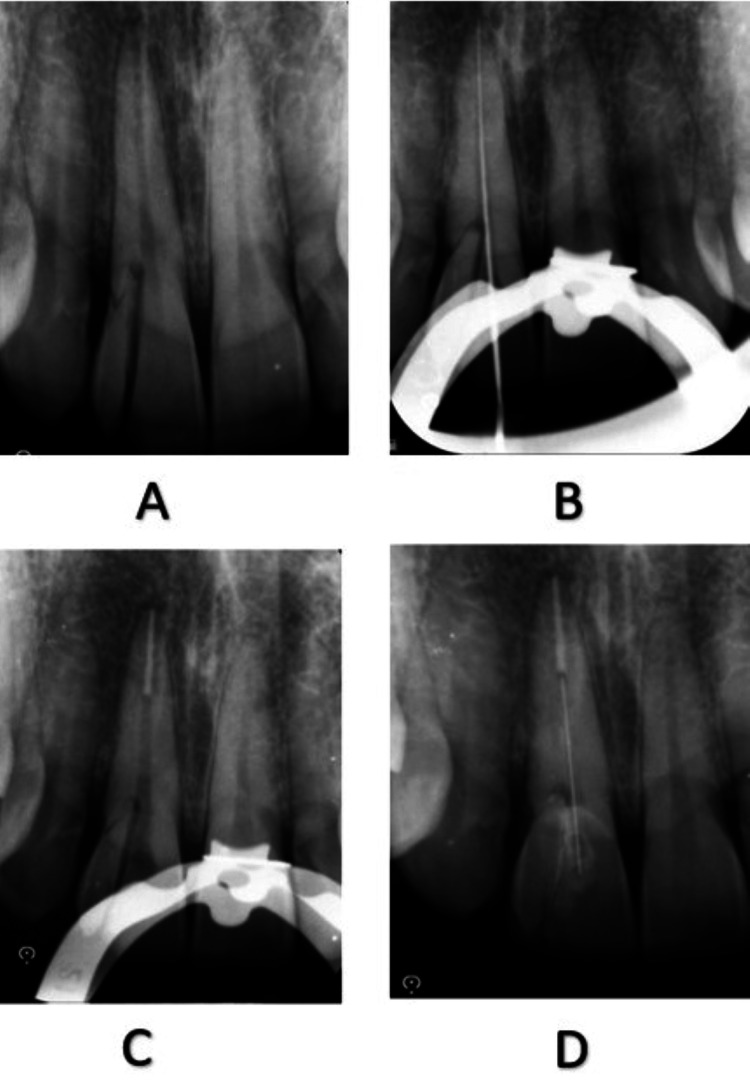
Intraoral periapical radiographs: (A) pre-operative radiograph of fractured 11; (B) working length determination; (C) sectional obturation; (D) post-operative radiograph after post placement and fragment reattachment

A 30-year-old male reported with a chief complaint of fracture in the upper front tooth, within 12 hours due to a fall from his vehicle. On clinical and radiographic examination, a vertical crown fracture was found extending subgingivally below the level of cementoenamel junction (Figures 11, 12A). There was no concomitant soft tissue damage and the probing depth was normal. An evaluation of the fractured segment was carried out and informed consent was obtained from the patient before the procedure.

Single-visit endodontic treatment was proposed to be carried out in 11. After local anesthesia, the fractured segments were temporarily stabilized with glass ionomer cement. Access opening was initiated and working length was confirmed with a radiograph (Figure 12B). Cleaning and shaping was done using 5.25% sodium hypochlorite as an irrigant; 5 mm of the apical seal was obtained with sectional obturation (Figure 12C).

**Figure 13:**
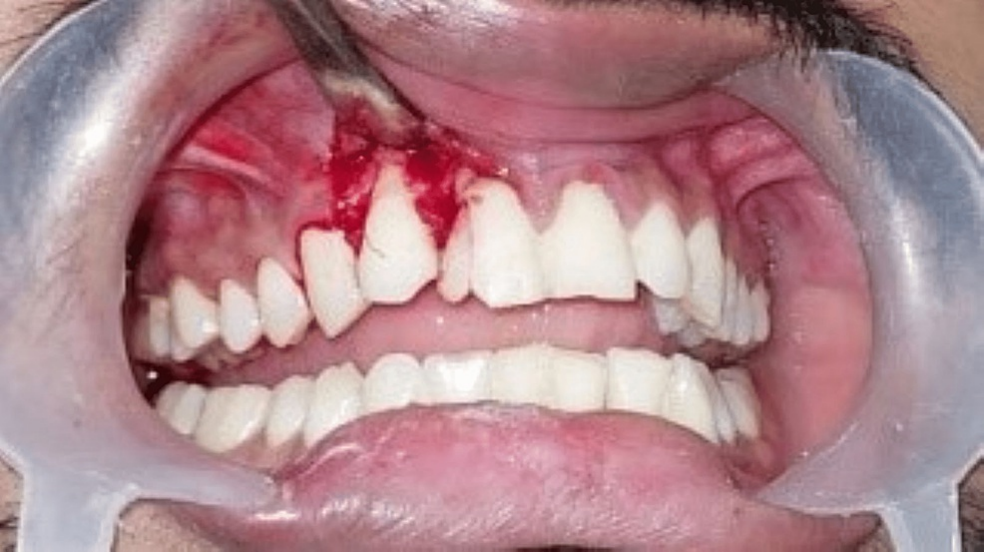
Elevation of the triangular flap prior to fragment removal in 11

Before removal of the fractured segment, a crevicular incision was made in 11 and 12 regions followed by vertical incision with a 15 BP blade in the distal aspect of the 12 region. Elevation of the flap was done to evaluate the extent of the fracture line. A triangular gingival flap was elevated and the fractured segment was removed with the help of tissue forceps (Figure 13). After achieving adequate access, the fracture line was seen not invading the biological space. The region was disinfected with povidone iodine solution. The fragment was extensively cleaned with 5.25% NaOCl to eliminate any pulpal tissue, and was placed in saline for storage. After achieving hemostasis, post space preparation was done with Peeso reamer size 2. The appropriately sized prefabricated fiber post of 1.1 mm diameter was selected.

**Figure 14:**
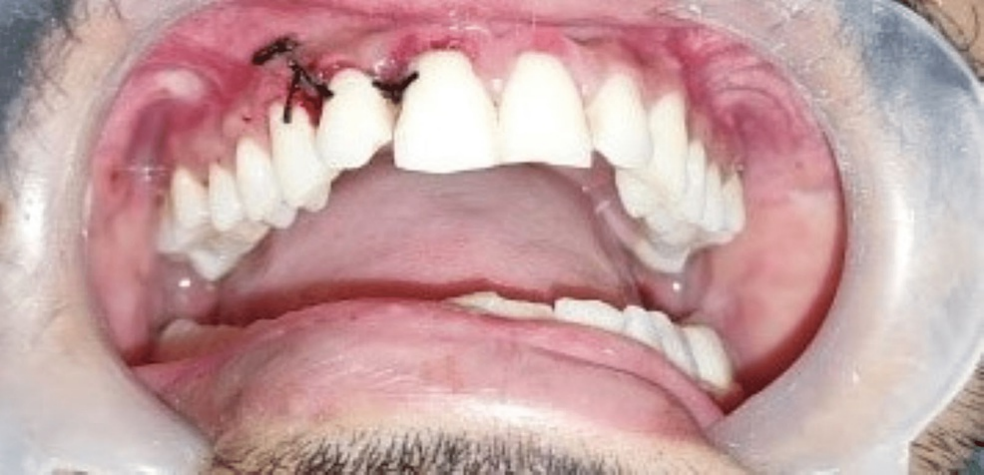
Post-operative clinical photograph after suturing

**Figure 15:**
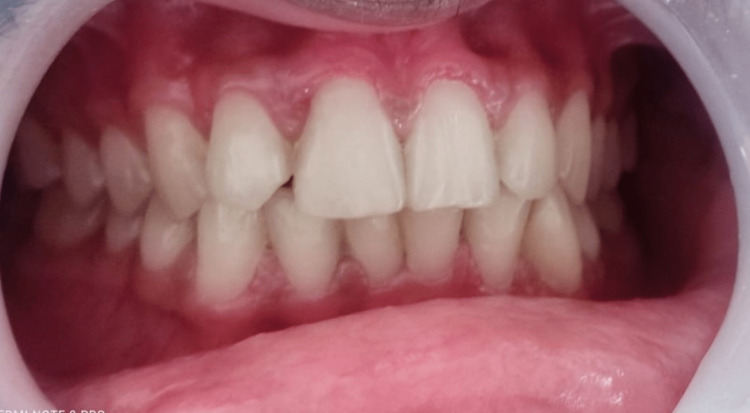
Post-operative clinical photograph after the three-month follow-up

Post space and fiber post were etched with the universal etchant for 15 seconds. Rinsing and gentle air drying was done. The application of adhesive was done on both the fiber post and etched surface. Dual cure cement was used to lute the post and curing was done for 40 seconds. The fragment was carefully placed on the tooth surface and luted with the composite. Light curing was done for about 40 seconds (Figure 12D). The remnant excess of the composite was removed and the tooth surface was polished. Later, the flap was repositioned and suturing was done (Figure 14). Final finishing and polishing of 11 was done. The occlusion was verified and the patient was given instructions to avoid excessive forces. The patient was reviewed at one, three and six months for follow-up (Figure 15).

## Discussion

Anterior tooth fracture is a common traumatic injury. It is of greater concern to the patient since it affects one's aesthetics. In such circumstances, management should be conservative in order to achieve a favorable outcome. The traditional methods to restore the fractured teeth are the use of partial coverage crowns, full coverage crowns, composite resin restorations and laminate veneers. But they have several drawbacks like cost, time, and the fact that they are not very conservative [9]. Chosack and Eidelmanin 1964 proposed that restoring the fractured teeth by reattaching the tooth fragments could be the best technique to restore the tooth's natural form, contour, surface texture, color and occlusal alignment [7]. According to Cavalleri and Zerman, the reattachment of fractured segments appears to have a superior and long-term prognosis compared to composite resin restorations [10].

Maintaining the tooth's natural aesthetic appearance along with providing sufficient bond strength requires proper hydration of the fractured segment [11]. Storage of the fractured segment in sterile saline at room temperature of 37°C can prevent the fragment from collapsing and dehydrating, thereby avoiding any dimensional change in the fragments [12]. Therefore, sterile isotonic saline was employed for hydration.

The selection of adhesive material is another important factor in the reattachment of tooth fragments. High bond strength is provided by adhesive systems between the fragments and the damaged tooth [13]. de Sousa et al. stated that the best method for reattaching tooth fragments was to use an adhesive system in combination with an intermediate composite [14]. According to Garcia et al., the most effective treatment depends upon the type of fracture and the degree of adaptation between the fragmented portions of the tooth [6].

An essential factor in re-restorability is the position of the fracture line that directly affects the prognosis of such teeth [5]. The process for reattachment would be simple if the fracture line is supra-gingival. However, a post retained crown or orthodontic extrusion may be required if the fracture location is subgingival or intraosseous. Alternative surgical procedures include elevation of the tissue flap, surgical crown lengthening, electrosurgery and gingivectomy for bonding of the fractured segment. According to Baratieri et al., minimal osteotomy and osteoplasty should be used during surgery whenever the fracture site invades the biologic width [15]. In the cases that were attempted, namely, cases 2 and 3, the fracture lines presented a favorable position for fragment reattachment. Proper contour and surface finishing of the subgingival restorations become necessary for better prognosis of the reattached teeth [16].

Since there were no radiographic signs of periapical infection, single-visit root canal treatment was performed. To our knowledge, there is no sufficient information to conclude if there are significant variations in the results obtained between single-visit and multiple-visit root canal treatments [17]. The treatment was completed in a single appointment as the fracture extent made it indispensable to reattach the fragments immediately, thereby preventing the risk of infection from interim restorations. Also, dehydration of the fractured segments and ingrowth of soft tissue can occur if treatment is delayed.

Extensive tooth damage and missing fragments require dental reinforcement with fiber posts followed by crown placement. Post systems are generally advised if the fracture is found involving two-thirds or more of the crown structure. In case 1, since there was enough crown structure, the fractured segment was stabilized following which endodontic treatment was done. Furthermore, composite build-up was done to reinforce aesthetics and function. Reattachment with a fiber post was proposed as the best treatment plan in cases 2 and 3. Traditionally, cast metal post and core are employed in these scenarios. However, due to their high elastic modulus, metal posts result in catastrophic root fractures [18]. Hence, the use of metal posts has declined reasonably. Thus, in cases 2 and 3, fiber post was used to support and retain the fractured segments after endodontic treatment.

The most recent versions of nonmetallic posts are made of ceramic or fiber-reinforced materials like carbon, glass, or quartz with an epoxy matrix. Fiber posts exhibit a similar modulus of elasticity to dentin, which produces strain similar to that of sound teeth [18]. The even distribution of stress to the remaining radicular dentin is another advantage of fiber posts. In addition to strengthening the teeth, the resin cement used for luting the fiber post aids in enhancing the bond strengths of the fractured segments and reduces the presence of voids [18,19]. Dual curing techniques were considered as the best options in these cases since they allow for complete polymerization in subgingival areas where complete penetration of the light is not possible [20]. Since eugenol-based sealants may prevent the setting of resin cements, resin sealers were employed for obturation of the teeth intended for restoration with glass fiber posts. In the cases discussed here, the protocol as mentioned by International Association of Dental Traumatology (IADT) guidelines was followed [21].

## Conclusions

The technique and materials used in fragment reattachment are determined by a plethora of factors. Reattaching tooth fragments is a conservative and quick way to satisfy a patient's aesthetic need while minimizing long-term complications. Utilizing fiber-reinforced posts allows for the creation of aesthetically pleasing restorations while also effectively preserving and strengthening the tooth structure. To achieve a good long-term prognosis, proper case selection, preoperative assessment and meticulous planning are mandatory. In our case series, three different approaches for the management of complex crown fractures were undertaken.
